# General Practitioners' Perceptions of the Use of Wearable Electronic Health Monitoring Devices: Qualitative Analysis of Risks and Benefits

**DOI:** 10.2196/23896

**Published:** 2021-08-09

**Authors:** Lucia Volpato, María del Río Carral, Nicolas Senn, Marie Santiago Delefosse

**Affiliations:** 1 Research Centre for Psychology of Health, Aging and Sport Examination Institute of Psychology University of Lausanne Lausanne Switzerland; 2 Department of Family Medicine Centre for Primary Care and Public Health University of Lausanne Lausanne Switzerland

**Keywords:** mHealth, wearable devices, health wearables, activity trackers, health monitoring, self-tracking, general practitioners, mind maps, qualitative research, health psychology

## Abstract

**Background:**

The rapid diffusion of wearable electronic health monitoring devices (wearable devices or wearables) among lay populations shows that self-tracking and self-monitoring are pervasively expanding, while influencing health-related practices. General practitioners are confronted with this phenomenon, since they often are the expert-voice that patients will seek.

**Objective:**

This article aims to explore general practitioners’ perceptions of the role of wearable devices in family medicine and of their benefits, risks, and challenges associated with their use. It also explores their perceptions of the future development of these devices.

**Methods:**

Data were collected during a medical conference among 19 Swiss general practitioners through mind maps. Maps were first sketched at the conference and their content was later compared with notes and reports written during the conference, which allowed for further integration of information. This tool represents an innovative methodology in qualitative research that allows for time-efficient data collection and data analysis.

**Results:**

Data analysis highlighted that wearable devices were described as user-friendly, adaptable devices that could enable performance monitoring and support medical research. Benefits included support for patients’ empowerment and education, behavior change facilitation, better awareness of personal medical history and body functioning, efficient information transmission, and connection with the patient’s medical network; however, general practitioners were concerned by a lack of scientific validation, lack of clarity over data protection, and the risk of stakeholder-associated financial interests. Other perceived risks included the promotion of an overly medicalized health culture and the risk of supporting patients’ self-diagnosis and self-medication. General practitioners also feared increased pressure on their workload and a compromised doctor–patient relationship. Finally, they raised important questions that can guide wearables’ future design and development, highlighting a need for general practitioners and medical professionals to be involved in the process.

**Conclusions:**

Wearables play an increasingly central role in daily health-related practices, and general practitioners expressed a desire to become more involved in the development of such technologies. Described as useful information providers, wearables were generally positively perceived and did not seem to pose a threat to the doctor–patient relationship. However, general practitioners expressed their concern that wearables may fuel a self-monitoring logic, to the detriment of patients’ autonomy and overall well-being. While wearables can contribute to health promotion, it is crucial to clarify the logic underpinning the design of such devices. Through the analysis of group discussions, this study contributes to the existing literature by presenting general practitioners’ perceptions of wearable devices. This paper provides insight on general practitioners’ perception to be considered in the context of product development and marketing.

## Introduction

Over the last few years, the development of new health-related technologies has been particularly rapid and prosperous [1,2]. In particular, wearable electronic health monitoring devices (henceforth referred to as wearable devices or wearables) are designed to support health management by monitoring bodily vital signals, as well as tracking an individual’s activity and habits [3,4]. User-friendliness and gamification play an important role in the appealing and engaging design of wearable devices, which are often paired with mobile phone apps [5]. Their association with mobile phones is at the origin of the terms *mHealth* (or *mobile health*) and continuous connection with wireless devices has been associated with self-surveillance and self-tracking mentality [6]. Yet, the difference between technologies targeting lay populations and the ones designed to monitor specific medical conditions is not always clearly defined [3]. In both fields—health promotion and intervention—the logic underpinning wearable devices’ marketing aims to improve health by promoting behavior change through self-tracking, on the basis of feedback mechanisms [4,7,8].

More specifically, the marketing discourse on wearable devices is strongly based on the promise of benefits regarding personalized health management programs that claim to promote patient’s self-responsibility and autonomy [6] by fostering a less hierarchical relationship between the user and the health professional [7]. Furthermore, wearable manufacturing companies have been sponsoring large-scale medical studies [9,10] with little consideration of the consequences associated with the introduction of self-tracking devices in everyday life [11]. This adds further complexity to the picture because traditional health care systems risk being detoured by other—often profit-led—motives [11]. While it has been argued that wearable devices may contribute to fostering users’ autonomy [12], self-tracking has not always been associated with empowerment [13,14]. In fact, wearable use could hinder the autonomy of users, who would increasingly rely on these devices for daily, health-related decision making [4]. According to Andreassen and colleagues [15], feelings of domestication and resistance co-exist in the user–object relationship. Furthermore, wearable devices have been associated with high abandonment rates after only 6 months of use [16]. It is, therefore, clear that, beyond manufacturers’ promises, the concrete use of these technologies in the health sector is subject to negotiation processes that depend on complex dynamics regarding the object–user relationship and the doctor–patient relationship [17,18].

Activity trackers have affected how treatment, visits, and health management are established during health consultations [19]. Their growing presence may constitute a positive addition to the relationship between patients and their general practitioners (GPs), because the devices could support effective transmission of health-related information [19]. Historically, the introduction of technological devices has defined medical practices [20], and wearable devices are no exception. The notion of the Quantified Self—associated with activity trackers—is pervasively shaping health norms through self-surveillance across life domains [21]. Moreover, the contemporary trend of healthism, which values individual responsibility and surveillance in health management, has expanded over the past decades [22]. This societal discourse constitutes a fertile ground for the production and marketing of wearables.

GPs are an inherent part of the digital health revolution, given their role as health experts, citizens, and sometimes, wearable consumers [23]. The intention to adopt activity trackers and recommend their use are shaped by views and attitudes that individuals may have toward such technologies [24,25]. In light of these findings, it is necessary to further explore perception and views on wearables among GPs, because such devices may change existing medical practices and contribute to shape new ones. This is particularly relevant within the Swiss context, where, similarly to other European countries [26,27], GPs are in the front-line when patients access the health system. In this sense, often GPs are the first health professionals to interact with many wearable device users.

Some studies have investigated health professionals’ attitudes toward technologies that are specific to chronic health conditions, such as epilepsy [28], asthma [29], arthritis [30], and other chronic diseases [31]. Another body of literature on GPs’ perspectives has examined their experience with a wide array of eHealth innovations, beyond the specific use of wearables [32]. With respect to wearables, few studies have explored GPs’ attitudes with a set of predefined topics using individual semistructured interviews [33,34] and web-based surveys [19]. We further investigated GPs’ perspectives and contribute to the existing literature on the role of wearables in family medicine. We aimed to explore how GPs perceive wearable devices—both for health promotion and clinical use—in the context of their medical practice, by focusing on perceived benefits and risks. To do so, we used an innovative qualitative methodology with mind maps to analyze group discussions that took place during a medical conference on family medicine. Mind maps have been described as being particularly suitable for analyzing group discussions in the field of health care [35]. We discuss salient elements to consider in the future development of these technologies.

## Methods

### Research Context and Sampling

This study’s aims were defined by the authors in collaboration with health psychologists and physicians working at the University of Lausanne, as well as GPs working in the French-speaking regions of Switzerland. Data were collected in a symposium—New Technologies in Family Medicine—that took place in Switzerland in 2018, as part of a medical conference, mainly targeting GPs. Given the qualitative nature of our study, we aimed at in-depth understanding and contextualization of data, rather than generalization. For data collection and analysis, we followed the quality criteria for qualitative research defined in the field of health psychology [36,37].

We used convenience sampling: 19 GPs (7 female and 12 male) working in family medicine in the French-speaking regions of Switzerland. Participants were informed about the symposium’s goal, involving the definition of potential research perspectives regarding the use of wearables in family medicine, based on their perception. GPs were formally informed that group discussions would be recorded for further analysis, and oral consent was obtained. Under Swiss ethical regulations, no written consent was required as no biomedical information was collected.

Regarding participants’ background on wearables use, the vast majority reported not having actively introduced them in their clinical practice and that any discussion on wearables was usually initiated by patients themselves. Cited examples included patients who monitored their menstrual cycle through apps and the tracking of physical activity through smartwatches. Only 2 GPs reported that they used mobile apps for sleep monitoring and for diagnostic procedures via symptom-input mechanisms. It was highlighted that such apps were offered by official health providers. Some participants were familiar with such technologies through life experiences beyond their professional practice as GPs. With respect to their personal use, 1 GP reported using a smartwatch for performance monitoring during sports training. In contrast, another participant reported deactivating all tracking functions on their mobile device because of mistrust of the app’s use of personal information.

### Group Discussions

GPs were enrolled in group discussions on smartwatches, wearable devices, and health apps. These topics had been previously defined, so that participants could join any group discussion, based on their personal interests. Each group (average of 6 participants per group) was moderated by 1 health psychologist and 1 GP. Discussions lasted approximately 1 hour and were audiorecorded.

In each group, participants were invited to briefly present themselves and were informed that the discussions were going to address the role of multiple mHealth technologies in family medicine. The 2 moderators introduced a brief explanation of the specific discussion topic (either smartwatches, wearable devices, or health apps, each discussed within a different group). These 3 groups of technologies were chosen for their high interconnectedness and interdependence within the broad category of mHealth [6]. For instance, smartwatches may be considered a wearable device category and are often supported by a smartphone app for data collection and analysis [5]. Participants were asked to discuss the following questions within each group: (1) What is the role of such technologies within your practice, according to your experience? (2) What risks and benefits do you identify in relation to such technologies? (3) Which challenges would you associate to the concrete use in your professional practice?

While the differences with other methods of data collection (ie, group interviews or focus groups) may be subtle, group discussions are less bound to structured interview guides and the emerging discussion topics often result from the interactions among group members, rather than from detailed predetermined questions grid [38]. Group discussions are also particularly suited for data collection among individuals who belong to the same group, for example, a professional category [38,39]. Moreover, the role of the moderators in group discussions is to provide topics to stimulate interactions among participants in a nondirective way [38].

To facilitate participants’ interactions, moderators took part in the discussions and summarized the material produced from their group. Summaries were approved and validated by participants of each group, resulting in specific descriptive reports [40-42]. Participant validation has shown to be a critical stage of qualitative research, because it provides more solidity and pertinence to the collected data [37].

### Mind Maps

The potential of mind maps has been recently underlined for their use as research methods for data collection and analysis in the field of health [35]. A mind map can be defined as “a diagram used to represent concepts, ideas or tasks linked to and arranged radially around a central key word or idea [35].” Mind maps present information in a hierarchical way [43] through a synthetic visually engaging format [44]. Beyond their use in data collection, they can facilitate the data analysis by identifying and representing thematic and conceptual patterns in a nonlinear form [45], while showing associations between ideas and topics [43]. During the symposium, an overt participant–observant researcher (the main author) circulated among the different discussion groups, taking notes of part of the ongoing discussions, and sketching preliminary mind maps. Through participant observation, further notes were taken to identify the links between the raised concepts and capture the contextual dimensions of verbal exchanges [46].

Inspired by Burgess-Allen and Owen-Smith [35], we considered a separate mind map for each of the themes used for the 3 groups canvases: benefits, risks, and some insights for the future. In addition, mind maps drawn during the group discussions revealed a recurring substructure in the discussion of the themes: doctor–patient relationship, patient–device relationship and GPs’ broad concerns, and final mind maps reflected this structure. The content of each mind map was then assembled inductively, and narrative contents were systematically compared by assessing their semantic similarities and differences. The proceedings and the audiorecordings from the group discussions facilitated integration of any missing relevant information from the preliminary notes and mind map drafting. This was particularly helpful to confirm the accuracy of the qualitative material and the mind map analysis. Finally, the 3 mind maps were compared to one another to identify common issues raised across group discussions regarding the potential benefits and risks, as well as, some insights for the future of wearable devices. This technique allowed for data analysis according to a theme-categories-subcategories structure, analogous to inductive thematic analysis, where mind mapping is a preliminary stage [47]. In this study, mind maps were first sketched on paper for conceptualization purposes and were later digitally reproduced with FreeMind software (version 1.0.1).

## Results

### General

**Figure 1:**
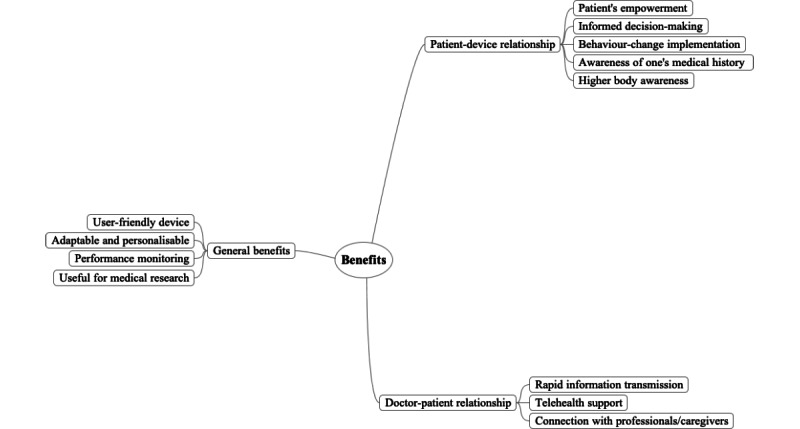
Mind map presenting the benefits that general practitioners associate with wearable use.

**Figure 2:**
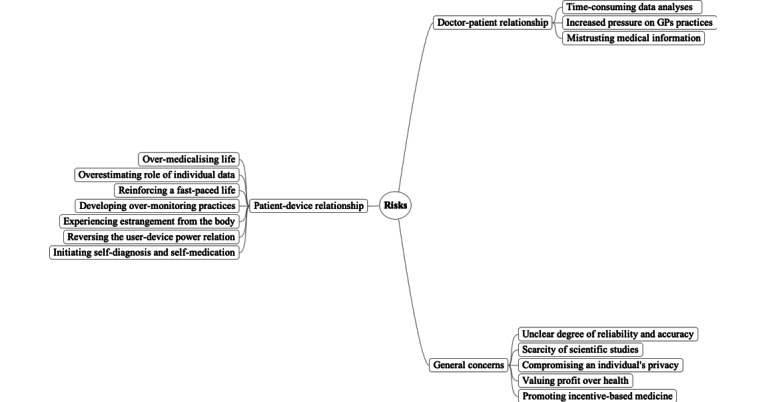
Mind map presenting the risks that general practitioners associate with wearable use.

**Figure 3:**
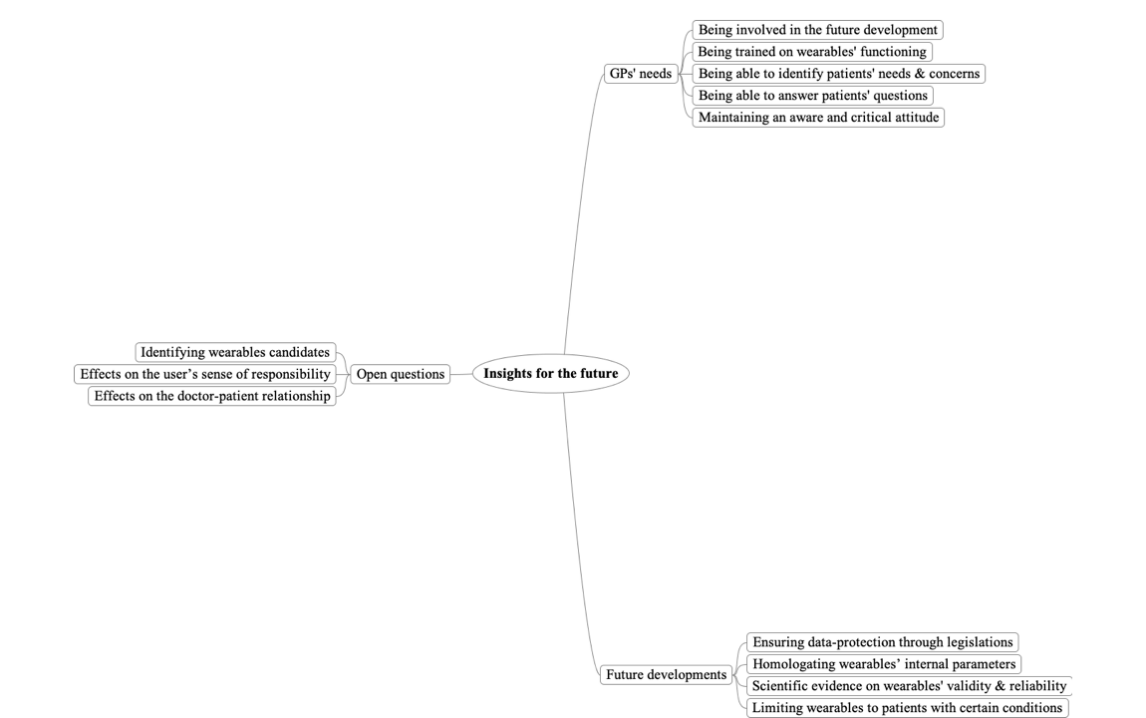
Mind map presenting insights for the future of wearables according to general practitioners.

Regarding GPs’ perceptions of smartwatches, wearable devices, and health apps in family medicine, the first mind map (Figure 1) summarizes the perceived benefits of wearable devices. Here, participants used the conditional verb tense, which suggested that their arguments often applied to hypothetical scenarios and specific conditions. The second map (Figure 2) shows perceived risks that wearable devices usage and promotion may entail. The third map (Figure 3) presents insights that should be considered in the future production and use of wearables.

### Benefits That GPs Associate With Wearable Use

#### General Benefits Associated With Wearable Devices’ Characteristics

Overall, GPs positively evaluated wearable devices that were considered user-friendly to be used in a variety of health situations, thereby representing an attractive solution for different populations. According to GPs, wearable devices could be easily used by both patients and health professionals due to their simple, intuitive designs. In particular, doctors appreciated devices whose parameters can be easily adapted and personalized to fit a patient’s personal health characteristics. In fact, these features could enable a personalized approach to health management. Moreover, GPs considered that wearables could benefit those who wished to have regular feedback about their personal health and measure their physical activity through performance monitoring. According to GPs from the 3 group discussions, the widespread use of wearables could allow for large-sample data collection, which would be especially useful for medical research. In fact, a potential benefit concerned the strong statistical power that wearable devices could enable through research conducted among a high number of users.

#### Patient–Wearable Relationship

Wearable devices were also discussed in relation to patients’ empowerment. According to different participants across the 3 group discussions, wearables could help raise awareness among patients on their overall health condition. Due to the feedback and reward mechanisms that define certain devices, wearables could train users to make informed health decisions. In this sense, results reveal that wearables may enhance patient’s self-responsibility and be a concrete partner for health promotion. In fact, wearables were described as a potential mean for behavior change through the implementation of new health behaviors, through consistent self-management. As stated by a participant:

Smart watches could motivate people for being more active, because there’s a certain degree of satisfaction in seeing the [step] counting going up.

Moreover, some GPs asserted that wearables could help patients to keep track of their medical history and develop an unprecedented awareness regarding their own bodies. For instance, wearable users could establish links between their own feelings and the data provided by the device. In their views, wearables may, in fact, concretely support self-education.

#### GP–Patient Relationship

Wearables were described as potential health care partners in the light of the rapid information-transmission processes that such devices enable, allowing patients to have a more central role in their health management. Furthermore, the time-efficient data exchange between patients and health professionals could be beneficial for the management of certain health conditions, such as epilepsy or cardiac diseases. These health conditions were mentioned given the capacity of wearables to transfer data in real time. From GPs’ views, this represented a helpful feature to prevent seizures or enable screening procedures. Wearables were also perceived as potential allies for telehealth, since these devices could help GPs reach patients living in geographically remote areas.

One GP affirmed:

We don’t need to make a trip to their place if they can measure themselves blood tension, glycemia, the heartrate at home and then transfer information via the internet.

This was considered a particularly important aspect in the Swiss context, where, due to alpine geography, certain patients may need to travel long distances to receive medical care. Wearables were described as a possible means of connection within the health system between different professionals and caregivers, as well as a useful solution for those who do not have family or social support in daily health care.

### Risks That GPs Associate With Wearable Use

#### General Risks Associated With Wearable Devices’ Characteristics

Although wearables’ potential advantages were thoroughly discussed, the debate raised several potential risks linked to their use. For instance, participants expressed fears regarding the unclear degree of reliability and accuracy of commercial wearables that are increasingly available. GPs shared their professional experience in stressing that devices that are able to accurately record biometrics are often more expensive and more complex. Therefore, their use requires specific training and an understanding of data collection. Furthermore, GPs highlighted the scarcity of scientific studies on wearables’ validity and reliability. To them, this represented an obstacle that impeded them from actively promoting the use of instruments that are not supported by scientific evidence.

Furthermore, the lack of accurate information regarding the management of biomedical data by manufacturers was considered to be a serious danger for patients. The risk of compromising an individual’s privacy was a major concern with respect to these technologies since, as affirmed by a participant:

Third-party use of personal data is still very poorly regulated.

In an era where personal data is becoming widely commodified, several industries can profit from wearable use without being genuinely concerned by users’ health. According to GPs, the promotion of wearables could thus imply that financial profit is valued over health.

The role of health insurance companies and their possible relation with the wearable device industry were also considered in group discussions, since the Swiss health system relies on compulsory private not-for-profit health insurance companies. According to some participants, the collaboration between the 2 stakeholders could encourage the development of incentive-based medicine rationalities, that is, of a health philosophy, by which patient behavior could be rewarded or punished by insurance companies as a consequence of the degree of behavior compliance determined by the wearable’s design. For instance, health insurance companies could be inclined to reward so-called good users for having achieved the health aims set by the device or punish so-called bad users who have failed to do so. This mechanism deserves better attention, because it may also potentially reinforce health inequalities from a socioeconomic perspective. These reflections raised a debate on which ethical principles should underpin family medicine, as well as, on the rights and responsibilities of each actor.

#### Patient–Wearable Relationship

Regarding user–device relationship, GPs argued that continuous self-monitoring could stress an overmedicalization of life, generated by an excessive intellectualization of the user’s physical condition. From their perspective, the prioritization of self-monitoring practices in the field of health would inevitably confront users with the paradoxes of our culture: while health-related practices are aimed at reducing stress in daily life by helping users to slow their pace, wearables would be the symbol of a society that values rapid information exchange, and hence, would contribute to reinforcing a fast-paced life. In this context, GPs raised the risk of overestimating the value and the role of individual data in coming to conclusions about a person’s general state of health. According to GPs, the continuous measurement of biomedical information appeared to be also potentially anxiety triggering. A participant feared that

People may end up spending more time preoccupying about their health instead of living.

Patients with apprehensive personalities could particularly risk developing overmonitoring practices, to the detriment of their mental health.

Regarding the level of trust toward certain devices, some participants feared that wearables would induce the users to gradually feel estranged from their body. In this sense, wearables could provide digital information that does not correspond to the users’ subjective perceptions on their own body and health. This mismatch between the wearable’s feedback and the embodied sensations could induce the users to mistrust their subjective perception and thus feel disconnected from their own body. According to GPs, this risk would also interfere with the principle of patient autonomy, whose appraisal of their own body would therefore mainly depend on the wearable verdict instead of their own perception. In this scenario, the patient and the caregiver would need to invest even more resources to set up a process of bodily re-appropriation. From the participants’ view, these risks would result in a reversed power relationship with the device that could be dangerous and that should be avoided. A participant feared a

Very likely tendency towards over-training during a sport session

while seeking positive feedback from an activity tracker. An important element of the debate concerned wearable data interpretation. Participants agreed on the fact that, given a decrease of exchanges between users and health professionals, the former would be confronted to increased uncertainties regarding the interpretation of their personal physiological values, which is considered to be as dangerous for a user’s health. In fact, on the basis of the wearable data, patients could be tempted with self-diagnosis or self-medication solutions, something that ought to be avoided, especially when medical expertise is essential.

#### GP–Patient Relationship

In the light of the increased production of patient-specific medical information, participants highlighted the risk of devoting their working hours to time-consuming data analyses.

We can collect plenty of data, but then what will do about them?

wondered a participant. In fact, the instantaneous nature data transmission could intensify the expectation of an immediate reply from health professionals, which would amplify the pressure on GPs’ daily practices, without any verified benefit for concerned patients. Participants also expressed the danger of inconsistency between the information recorded by wearables and data provided by other devices measuring biometrics. This type of divergence could, in fact, entail a progressive mistrust on the part of patients regarding the information provided by other the medical instruments, and GPs may suffer from credibility loss.

### Insights for the Future of Wearables According to GPs

#### GPs’ Professional Needs

Participants expressed a sense of inevitability toward the introduction of wearable devices into contemporary medical practices within the Swiss context, regardless of the outcomes of current research in the field. In this sense, several GPs highlighted the urgent need, and their personal interest, to become more involved in the development of wearables. In their view, a synergy between producers and health professionals is necessary to enable the design of beneficial instruments. GPs also expressed the wish to receive training to better understand how these technologies work (especially concerning data collection and storage of information) and to be better informed on the news regarding the health-wearable market. GPs particularly valued the importance of understanding patients’ concerns, of identifying patients’ health needs, and of answering to their questions.

When worried patients come [to the consultation] with such instruments, what do we do? We have to give them an answer,

stated a participant. They also expressed the importance to them as professionals to be aware and to remain critical of the usefulness these devices in health care.

#### Future Developments

Overall, participants from the 3 group discussions raised the urgent need for firm legislation to guide future design, production, and marketing of wearables. In particular, they showed a high degree of mistrust regarding the confidentiality of their patients’ data that wearable companies could guarantee. To them, a priority for the future would therefore, be legislation to ensure data protection, as well as an overt policy to safeguard the rights of users, because the latter are in a vulnerable position within the health care system. Furthermore, the legislation should also concern the homologation of wearables’ internal parameters. Alongside scientific research, these measures could help to produce more valid and reliable devices for health self-management. With respect to the role that wearables should have, participants expressed the vision of wearables as partners that could help improve the care of patients. As emphasized by certain participants, well-oriented and accurate feedback is central to medical practice, because it can facilitate the learning process. In this sense, the analyses showed that wearables would not only be a tool for information transmission but could solidify partners in the promotion of behavior change. Moreover, according to the participants, the use of wearables should be limited to patients suffering from certain health conditions (although no examples were explicitly given during the symposium), instead of monitoring healthy people. In this sense, health would be achieved by conducting a digital-free, slow-paced life, where the person is not dependent on self-tracking devices.

#### Open Questions

According to the participants, several questions remained unanswered yet would be worth exploring. For instance, some GPs wondered how to assess which patients would best benefit from wearable use, and on the contrary, which patients would feel disconnected from their own body, that is, lose their personal autonomy toward the interpretation of their own embodied feelings. In this sense, a participant asserted that

To be useful, such products could be adapted to the patient’s profile in the future.

GPs also wished to know how wearables may affect user’s sense of responsibility. More generally GPs also raised the following issue: How will health wearables affect the GP–patient relationship? These questions closed the debates across groups, highlighting the need of additional analyses before establishing any further statements on the role of health wearables for our contemporary societies.

## Discussion

### Principal Findings and Comparison With Prior Work

GPs play a crucial role in the health care system by promoting and prescribing specific health practices. We aimed to explore their perceptions on wearables in the context of family medicine, by directly addressing the risks and benefits associated with these technologies and reflecting on possible future developments in the field of health care. The methodology adopted in this research was qualitative. This perspective was particularly suited to generate exploratory and contextualized knowledge. While group discussions allowed us to capture GPs’ views throughout their spontaneous interactions, mind maps enabled an iterative and efficient process of data collection and analysis given our research setting [35].

### Wearables as Information Providers

The effects of digital health wearables on the doctor–patient relationship appeared to be both beneficial and risky, highlighting their ambiguous potential. While wearables were viewed as suitable for information transmission, coordination, and general illness management, GPs also feared that these technologies would put increased pressure on their role and expertise as health professionals. Indeed, GPs anticipated longer consultations that would be dedicated to data analysis and data interpretation stemming from patients’ wearables. From this perspective, our results confirm those of recent studies showing that wearables are considered to be particularly useful for information-transmission and general illness management but that time-consuming data interpretation continue to be important concerns among health professionals [19,34,48]. With respect to digital information, GPs also expressed their concern regarding product reliability and patients’ data protection. As recent studies have argued, developers need to consider these key issues when designing health-monitoring technologies [19,48,49].

Participants perceived wearables as user-friendly devices that could foster patients’ empowerment and support them throughout behavior change processes [33,34]. However, the use of wearables for patient education and empowerment has also been associated with a patronizing view of the doctor–patient relationship [6]. Preventing such repercussions represents a concrete challenge faced by research in the health sector. In this context, wearable use can be envisaged in relation to the concept of continuity in health care, defined as informational, relational, or management related [50]. In this sense, wearables constitute tools that can positively contribute to ensuring informational and health management continuity. Nevertheless, these tools alone may not be able to support the multifaceted relationship continuity between the doctor and the patient and would hence need to be adapted.

### Self-tracking: A Catalyst for Healthism

GPs were also concerned about the role that wearables could play in patients’ everyday lives outside medical consultations. For instance, GPs highlighted the potential risk of promoting a dominant social discourse or life-philosophy, where self-tracking and self-monitoring become practices that are encouraged, even among individuals who are healthy or who do not suffer from specific health conditions. This overmedicalization of life can be compared to what Gabriels and colleagues [33] have coined as *entertainment medicine*, where self-tracking devices become responsible for producing “medically unnecessary data that belong more to the fitness or wellness than to the medical realm.” Echoing past literature [32], GPs stressed the importance of understanding patients’ needs in order to address their concerns more effectively.

More generally, self-tracking in the medical field has been previously argued to have culturally and structurally transformed the ways in which health-related practices are being defined [15]. In this sense, an important contribution of our findings to the debate is GPs’ strong resistance to incentive-based medicine, in which healthy behaviors are implemented within a reward versus punishment mechanism. This posture contests 2 aspects. The first is with respect to the global trend across stakeholders to collect information produced by wearable devices for financial purposes [51], which causes ethical concerns to be raised by GPs. The second refers to the philosophical and pedagogical premises underpinning incentive-based medicine. In GPs’ views, this type of medicine contrasts with the value of patient autonomy and risks to promoting an undesirable obsessive compliance with health standards set by wearables. In the contemporary dominant culture of healthism that values self-management [22], this risk becomes increasingly important. Through subtle imperatives, wearables may indeed respond to patients’ intention to take control over their own health [21], while simultaneously triggering feelings of apprehension and self-inadequacy. GPs’ intentions of promoting patient autonomy emphasizes the urgent need to develop alternative approaches in health care that can facilitate behavior change. Indeed, as in the case of other social practices, health practices are subject to ambiguity, contradictions, and ultimately, continuous change [18]. We argue that these premises should be considered in the design of wearable technologies.

### Future Perspectives

The rapid expansion of wearables has entailed changes that remain unchallenged regarding their social, psychological and cultural implications for individual and public perceptions of health within our Western societies dominated by healthism [22]. In this sense, it is essential to clarify the rationale underpinning the development and marketing of such devices, whose extensive use may not necessarily be desirable from a GP’s perspective. A clear legal frame guiding the production and distribution of wearables for medical usage might help guide the effectiveness and clinical safety for users and health professionals. For instance, the concept of Health Technology Assessment [52] offers a useful illustration of how this frame could be conceptualized. This study calls for future research to deconstruct and analyze the logic behind the conceptualization, development, and use of health wearables, from the perspective of health professionals, users, and technology developers. In this context, it would be interesting to compare these results with patients’ views, in order to identify possible differences, with an aim toward better integration of wearables in general medical practice. Indeed, our study confirms the necessity for researchers and developers to question the values and logic guiding wearable design.

### Limitations

This study is not exempt from limitations. Given its exploratory nature, our qualitative results require further analysis regarding other contexts and methodologies. Moreover, while appropriate to our research setting, mind maps allow limited in-depth data analysis compared to other qualitative methods [35]. In addition, the visually synthetic characteristic of mind maps does not allow for data saturation claims and does not allow the integration of specific details. Rather, mind maps constitute an exploratory step in research that can be complemented by other techniques [47]. Nonetheless, this method is useful to develop hypotheses that can be tested in future research.

### Conclusion

This study found that GPs are willing to be more actively engaged as collaborators in the design, development, and promotion of wearables, alongside producers and end-users. Our research contributes to broadening current understanding of wearables and self-tracking technologies in the field of family medicine, by emphasizing the role of wearables as key information providers. Indeed, GPs are neither passive spectators of—nor opponents to—digital health developments, which are perceived to be increasingly more important and inevitable. In spite of the important role of wearables, this study underlined the irreplaceable character of the doctor–patient relationship, which remains a central dimension in family medicine. GPs manifested their opposition to the logic of self-monitoring that GPs considered to have a negative impact on patients’ global well-being and autonomy. Regarding research perspectives, it seems crucial to reflect upon the definition of health that is being shaped by wearables and similar self-tracking technologies. These perspectives would enable an informed comparison across main actors in health care and contribute to collective coordinated efforts to improve individual and public health while reducing health-related costs.

## Abbreviations

GP: general practitioner
mHealth: mobile health
